# A multi-step sequential pretreatment for boosting biomass conversion

**DOI:** 10.3389/fbioe.2026.1963128

**Published:** 2026-10-06

**Authors:** Weikang Teng, Hai Cheng, Yunni Zhan, Huiyang Bian, Fangmin Liang, Tongqi Yuan, Chen Huang, Guigan Fang, Hongbin Li, Xuelian Zhou

**Affiliations:** 1 State Key Laboratory for Development and Utilization of Forest Food Resources, Institute of Chemical Industry of Forest Products, Chinese Academy of Forestry, Nanjing, China; 2 Beijing Key Laboratory of Lignocellulosic Chemistry, Beijing Forestry University, Beijing, China; 3 International Innovation Center for Forest Chemicals and Materials, Jiangsu Co-Innovation Center of Efficient Processing and Utilization of Forest Resources, Nanjing Forestry University, Nanjing, China; 4 Key Lab of Biomass Energy and Material, Jiangsu Province, Key Lab of Chemical Engineering of Forest Products, National Forestry and Grassland Administration, National Engineering Research Center of Low-Carbon Processing and Utilization of Forest Biomass, Nanjing, China

**Keywords:** antioxidant activity, deep eutectic solvents, enzymatic hydrolysis, lignin, poplar

## Abstract

This study aims to overcome the structural recalcitrance of lignocellulosic biomass and improve the economic viability of biorefineries. A multi-step sequential pretreatment strategy combining hydrothermal pretreatment, twin-screw extrusion, and deep eutectic solvent (DES) treatments was developed to effectively deconstruct poplar biomass while recovering high-quality lignin, xylooligosaccharides (XOS), and fermentable sugars.The results demonstrated that this integrated approach achieved exceptional pretreatment performance, with 89.7% delignification and 85.3% xylan removal. The strategy enabled near-complete cellulose enzymatic hydrolysis at a low enzyme loading (15 FPU/g) and achieved a 45.7% XOS yield. Furthermore, the process yielded high-purity lignin (99.9%) with an 81.5% recovery rate,characterized by reduced molecular weight, enhanced antioxidant activity (IC_50_ = 56.16 mg/L), and a high phenolic hydroxyl content (2.81 mmol/g).This sequential pretreatment strategy exhibits significant potential for the integrated valorization of poplar biomass into fermentable monosaccharides, functional oligosaccharides, and antioxidant lignin derivatives, advancing the sustainability of lignocellulosic biorefineries.

## 1 Introduction

Biomass represents one of nature’s most abundant renewable resources, with vast quantities of agricultural and forestry residues serving as significant sources. The biorefining of these residues into chemicals and energy is not only a vital component of strategies aimed at replacing fossil fuels but also a key approach to mitigating environmental pollution caused by burning, while simultaneously achieving resource utilization and alleviating energy supply conflicts (Huang et al., 2020). Beyond the production of bio-chemicals and bioenergy, cellulose derived from lignocellulosic feedstocks can be processed into cellulose nanofibrils for advanced functional applications. Cellulose nanofibril-based composite films coupled with MXene and chitosan can achieve excellent flexibility and flame-retardant properties (Li et al., 2022). Meanwhile, bacterial-cellulose-based porous nanocomposites also exhibit great promise for high-performance wearable electronic devices (Guan and Guo, 2021). Lignocellulosic biomass possesses a complex structure primarily composed of cellulose, hemicellulose, and lignin. The diverse chemical bonds among these components—including ether, ester, and carbon-carbon bonds—significantly influence the conversion and utilization of lignocellulosic biomass (Yu et al., 2022). Consequently, pretreatment methods are necessary to overcome the inherent recalcitrance of lignocellulosic resources for subsequent high-value utilization (Mqoni et al., 2026). Traditional pretreatment approaches—such as acid treatment, alkali treatment, and steam explosion—can partially achieve this separation; however, each method exhibits certain limitations that constrain its further development and application (Novia et al., 2026).

Hydrothermal pretreatment, as an environmentally friendly and economical method, uses only water as the sole reaction solvent and exhibits excellent selectivity for hemicellulose removal, which shows great potential for industrialization (Gong et al., 2022). It not only enhances the saccharification properties of lignocellulosic biomass but also enables highly selective removal of hemicellulose, yielding products such as xylooligosaccharides (XOS), monosaccharides, and furfural (Feng et al., 2024; Zhao et al., 2022). However, although hydrothermal pretreatment can cleave lignin-hemicellulose linkages (benzyl ester, benzyl ether, and phenyl glycosidic bonds) via acidic media and remove most hemicellulose, its ability to remove lignin is poor. Furthermore, under high-temperature conditions, lignin redeposition further reduce the enzymatic accessibility of substrate cellulose, significantly hindering cellulase action on cellulose (Li et al., 2025). In recent years, deep eutectic solvents (DES) have been recognized as green solvents for lignin extraction, offering advantages such as good biodegradability and low cost (Xu et al., 2021; Hong et al., 2020; Chen et al., 2020; Wang et al., 2026). DESs consist of hydrogen bond donors (quaternary ammonium salts such as choline chloride) and hydrogen bond acceptors (polyols, carboxylic acids, amides) (Busato et al., 2021). Chen et al. (2023) pretreated sugarcane bagasse with betaine/lactic acid. The results showed that as the molar ratio increased from 1:1 to 1:4, the lignin removal rate rose from 36.7% to 50.3%. Balasubramanian and Venkatachalam (2023) efficiently extracted lignin from rice husks using a recyclable hydrogen bond donor (*p*-toluenesulfonic acid) and achieved an extraction rate of 83%. However, the limited utilization of hemicellulose during DES pretreatment hinders overall economic viability.

Single pretreatment methods exhibit limited fractionation efficacy. Integrating multiple pretreatment approaches can leverage their respective advantages, achieving synergistic effects for efficient decomposition and conversion of lignocellulosic biomass (Long et al., 2025; Bevilaqua et al., 2026; Bezza et al., 2026). Currently, numerous synergistic pretreatments have demonstrated outstanding results. Ma et al. (2021) used pulverized and sieved poplar wood as feedstock, applying hydrothermal synergistic DES pretreatment. The results showed that under hydrothermal pretreatment at 140 °C for 60 min, the XOS yield reached 30.97%. During the subsequent DES treatment, the saccharification efficiency of the fiber substrate peaked at 96.33%, and the isolated lignin exhibited outstanding antioxidant properties. Wang et al. (2021) used crushed and sieved moso bamboo as feedstock and conducted hydrothermal treatment, which yielded abundant monosaccharides. Subsequent treatment of the fibrous residue with a choline chloride/lactic acid system at 140 °C for 6 h achieved a lignin recovery of 86.9% and a maximum purity of 99.49%. Although these prior studies achieved efficient fractionation and utilization of lignocellulosic components, the mechanical pulverization and pretreatment processes consume significant energy, hindering large-scale industrial application.

To further enhance XOS and lignin yields for scalable application, this study investigated poplar subjected to various pretreatment systems: twin-screw extrusion, hydrothermal pretreatment, DES pretreatment, and multi-step sequential pretreatment. The research examined the fractionation efficiency of lignocellulose, physicochemical properties of pretreated substrates and lignin, and the effects of multi-step sequential pretreatment on saccharification and lignin antioxidant capacity. Twin-screw extrusion pretreatment subjects the feedstock to intense shear forces and mechanical friction, significantly disrupting cell wall structures, increasing fragmentation, and creating more pore structures. This substantially improves enzyme accessibility to the substrate fibers while partially removing surface lignin deposits formed during hydrothermal pretreatment, thereby reducing their negative impact on enzymatic hydrolysis. Finally, synergistic removal and recovery of substantial lignin using a DES system provides a methodological reference for achieving comprehensive utilization of poplar components in large-scale industrial applications.

## 2 Materials and methods

### 2.1 Experimental materials

Poplar chips (PL) were supplied by a paper mill in Shandong province, China. PL was mixed with a specific amount of water and processed through twin-screw extrusion and crushing. The resulting material (PL-E) was air-dried and stored in sealed bags for later use. Choline chloride, *p*-toluenesulfonic acid, and 1,4-butanediol (AR) were purchased from Macklin Biochemical Technology (Shanghai) Co. Ltd. CTec 2 cellulase (248 FPU/g cellulose) and X2753 xylanase (3490 U/g) were purchased from Sigma-Aldrich Co. LTD (Shanghai).

### 2.2 Pretreatment

#### 2.2.1 Twin-screw extrusion and hydrothermal pretreatment

Raw poplar chips with a nominal size of 20–30 mm in length and 2–5 mm in thickness were used as feedstock. The feed moisture content was adjusted to ∼50 wt% before extrusion. In the twin-screw extrusion, the raw material was squeezed in two squeeze area with outlet diameter of 24 mm for Zone I and 20 mm for Zone II (Jiangsu Jinwo Machinery Co., Ltd, Jiangsu, China). The screw rotation speed was set at 200 rpm, and the feeding rate was fixed at 3.0 kg/h on a dry basis. The material was processed in one single pass. No shaping die was installed at the barrel end, and extrudates were discharged directly as fragmented fibrous materials. The screw shaft was assembled with conveying elements and kneading blocks along the barrel. No external barrel heating was applied during extrusion, and the process relied on frictional heat generated by mechanical shear. The nominal throughput was approximately 3.0 kg/h under these conditions. The material residence time was not directly measured and was estimated to be 30–45 s. Specific mechanical energy was not quantified online due to the absence of an integrated torque sensor. The extruded material was collected for subsequent pretreatment.

PL were subjected to hydrothermal pretreatment at a solid-liquid ratio of 1:5 °C and 170 °C for 90 min. The pretreated liquor and pretreated residues (PL-P) were obtained by filtration through filter bags.

#### 2.2.2 DES pretreatment

Choline chloride, p-toluenesulfonic acid monohydrate (AR), and 1,4-butanediol (AR) were purchased from Macklin Biochemical Technology (Shanghai) Co., Ltd. Prior to use, choline chloride was dried under vacuum. The p-toluenesulfonic acid was used in its monohydrate form, which introduced trace amounts of water into the ternary DES system. The three components were mixed at a molar ratio of 1:1:1 and transferred to a three-necked flask. The mixture was continuously stirred with a magnetic stirrer in an oil bath at 80 °C for 1 h until a transparent, bubble-free, and homogeneous liquid was obtained. The freshly prepared DES was stored in a desiccator to prevent moisture absorption before use. Only freshly synthesized DES was employed in all pretreatment experiments.

For the pretreatment, 10 g (dried weight) of the material (PL, PL-E, PL-P, or PL-P-E) and 100 g of DES were reacted at 100 °C for 30 min. The reaction was terminated by adding 300 mL of an ethanol/water solution (1:1, v/v), followed by magnetic stirring for 2 h. The mixture was then separated by vacuum filtration to obtain the pretreatment liquor and the pretreated residues (PL-DES, PL-E-DES, PL-P-DES, and PL-P-E-DES, respectively), which were stored at 4 °C for later use. To precipitate the lignin, 600 mL of deionized water was added to the separated pretreatment liquor. The solid lignin was finally collected by centrifugation, washing, and freeze-drying, then weighed and stored in a desiccator.

### 2.3 Enzymatic hydrolysis

Pretreated residues (0.5 g) were accurately weighed into a 150 mL flask. The enzymatic hydrolysis was performed in a total volume of 50 mL. Cellulase and xylanase were added at dosages of 15 FPU/g cellulose and 300 U/g xylan, respectively. To adjust the pH to 4.8, 2.5 mL of 1 mol/L sodium acetate buffer was added. Moreover, 0.04 mL of 0.1 g/L tetracycline solution was added to inhibit microbial contamination, and 0.1 mL of Tween 80 was supplemented to reduce non-productive adsorption of enzymes onto residual lignin. The hydrolysis was carried out at 150 rpm and 50 °C for 72 h. At 24, 48, and 72 h, 1.0 mL aliquots of the hydrolysate were withdrawn, without replenishment of buffer. Each sample was heat-inactivated by boiling, centrifuged at 10,000 rpm for 5 min, and the supernatant was collected for determination of glucose and xylose concentrations. The cumulative volume loss caused by repeated sampling was taken into account and corrected in the yield calculation formulas.

The calculation methods for the enzymatic hydrolysis yields of glucan and xylan are as follows:

$$\text{Glucos}e\text{ yield}=\left(C_{g}\times 0.9\times \frac{v}{M_{g}}\right)\times 100\%$$

$$\text{Xylose yield}=\left(C_{x}\times 0.88\times \frac{v}{M_{x}}\right)\times 100\%$$

Where: Cg and Cx represent the glucose and xylose concentrations in the acid-hydrolyzed solution, g/L; V denotes the total volume of the enzymatic hydrolysis system, L; Mg and Mx denote the initial masses of glucan and xylan in the sample, g.

### 2.4 Antioxidant activity detection

The antioxidant activity of separated lignin samples was evaluated using DPPH as follows. A 0.2 mmol/L DPPH solution was prepared using anhydrous ethanol and stored at low temperature in the dark for later use. The lignin samples were dissolved in DMSO to prepare lignin solutions with concentration gradients of 0.05 g/L, 0.2 g/L, 0.4 g/L, 0.6 g/L, 0.8 g/L, and 1 g/L. For the reaction, 3 mL of the DPPH solution was mixed with lignin solutions in a brown centrifuge tube. After reacting in the dark for 30 min, measure the absorbance at a wavelength of 517 nm (Song et al., 2024). The calculation formula is as follows:

$$IP=\frac{A_{0}-A_{i}}{A_{0}}\times 100\%$$

Where: A_0_ is the DPPH absorbance, with anhydrous ethanol as the blank control; A_i_ is the absorbance of the lignin sample, with DMSO and anhydrous ethanol as blank controls.

### 2.5 Analysis and characterization

#### 2.5.1 Substrate characterization

Chemical composition of poplar before and after treatment (Zhu et al., 2025) was determined using a modified National Renewable Energy Laboratory (NREL) protocol employing a two-step acid hydrolysis procedure. 0.1 g dry sample was first hydrolyzed with 1 mL of 72% (w/w) sulfuric acid at 30 °C for 1 h, then dilute to the 4% acid concentration and autoclaved at 121 °C for 1 h. The hydrolysate was analyzed by high-performance liquid chromatography (Agilent 1260 II) equipped with a Bio-Rad Aminex HPX-87H column (55 °C) and a refractive index detector (35 °C), using 5 mM H_2_SO_4_ as mobile phase at 0.6 mL/min to quantify glucose and xylose. XOS content in the hydrothermal pretreated liquor was measured using a Dionex ICS-3000 high-performance anion-exchange chromatograph (HPAEC) equipped with a quadruple potential pulse anode detection (PAD) detector. The analytical column was a Carbon Pac PA 200, with a column temperature of 30 °C. A gradient elution was performed at a flow rate of 0.3 mL/min using a mobile phase consisting of 100 mmol/L NaOH and 500 mmol/L sodium acetate.

The surface microstructure of the pretreated residues was analyzed using a JSM-7600F scanning electron microscope (JEOL, Japan). Prior to testing, the surface underwent gold sputtering treatment.

The crystallinity of the raw and pretreated residues was determined using an Advanced D8 X-ray diffractometer (Bruker, Germany). A copper target X-ray source (λ = 0.15 nm) was employed with a test voltage of 40 kV and a current of 40 mA. The diffraction angle 2θ ranged from 10° to 40°, with a step size of 2°/min. The crystallinity index (CrI) was calculated using the following formula:

$$I_{C_{r}}=\frac{I_{002}-I_{am}}{I_{002}}\times 100\%$$

Where: I_002_ represents the maximum diffraction peak intensity of the 002 crystal plane (2θ ≈ 22.5°); I_am_ represents the diffraction peak intensity between the 110 crystal plane and the 002 crystal plane in the non-crystalline region (2θ ≈ 18°).

Fourier transform infrared spectroscopy (FTIR) was performed using an infrared spectrometer (Nikon iS50, USA) operating in transmission mode. The analysis involved 32 accumulated scans with a resolution of 4 cm^−1^ across the wavenumber range of 4,000 cm^−1^ to 400 cm^−1^. The areas of absorption of bands at 1,427 and 898 cm^-1^ were analysed for cellulose crystallinity index calculation according to the following equation:

$$LOI=A_{1427}/A_{898}$$

Where: LOI is lateral order index and A is the absorbance value of the corresponding band (Wang H et al., 2025).

#### 2.5.2 Lignin characterization

Two reference lignin samples, designated PL-MWL (from raw poplar) and PL-PE-MWL (from hydrothermally extrusion-pretreated residues), were isolated according to the classical Björkman milled wood lignin method with minor modifications. Briefly, each oven-dried sample was ground in a planetary ball mill at 360 rpm for 24 h with intermittent pauses. The milled powder was then extracted three times with 1,4-dioxane/deionized water (96:4, v/v) at room temperature under continuous stirring for 24 h in the dark, using fresh solvent each time. Then, the supernatant was collected and concentrated under reduced pressure at 40 °C. The concentrated lignin solution was precipitated by dropwise addition into 10 volumes of deionized water under stirring. The precipitated lignin was recovered by centrifugation, washed three times with deionized water, and freeze-dried.

The weight-average (Mw) and number-average (Mn) molecular weights of separated lignin was analyzed using a Shimadzu LC20A gel permeation chromatograph (Japan). Before analysis, 20 mg of sample was dissolved in 1 mL acetic anhydride/1 mL pyridine (1/1, v/v) for 24–72 h, then precipitated by dropwise addition into 10-fold volume of hydrochloric acid solution (pH < 2) with stirring. The precipitate was collected by centrifugation at 10,000 r/min for 10 min, washed three times with deionized water, and vacuum-dried at 40 °C to constant weight. Then, dissolved 4 mg of acetylated lignin in 2 mL chromatographic tetrahydrofuran and detected at 240 nm. The weight-average (Mw) and number-average (Mn) molecular weights of lignin samples were determined using an aWAT054475 column (Waters, USA). The system was operated at 35 °C with a flow rate of 1 mL/min, utilizing tetrahydrofuran (THF) as the mobile phase. Lignin samples were first acetylated with acetic anhydride and pyridine (1:1, v/v) prior to analysis. For measurement, 4.0 mg of acetylated lignin was dissolved in 2 mL of THF (2 mg/mL) and analyzed by GPC equipped with a UV detector at 240 nm.

Two-dimensional heteronuclear single quantum coherence nuclear magnetic resonance (2D-HSQC) and ^31^P NMR were conducted by a Bruker Ascend™ 600 MHz spectrometer. For 2D-HSQC analysis, the specific acquisition parameters were as follows: a spectral width of 166 ppm in F1 (^13^C) dimension with 256 data points, and 12 ppm in F2 (^1^H) dimension with 1024 data points. Additional parameters included a ^1^H–^13^C coupling constant (J_C-H_) of 145 Hz, a pulse delay of 1.0 s, and 128 accumulated scans to ensure sufficient signal-to-noise ratio for accurate spectral interpretation (Wang T et al., 2025). Semi-quantitative integration was performed using Bruker TopSpin 3.6 software. The integration regions were defined as follows: aromatic region (δC/δH 100–160 ppm/6.0–8.0 ppm) for aromatic C-H units, and side-chain region (δC/δH 50–90 ppm/2.5–6.0 ppm) for interunit linkages. The total integral of aromatic C-H signals was normalized to 100 Ar units, and the relative contents of lignin subunits and interunit linkages were calculated accordingly.

For ^31^P NMR analysis, anhydrous pyridine and deuterated chloroform were mixed in a 1.6:1 ratio to prepare a pyridine/chloroform solvent. An internal standard/relaxation agent solution was accurately prepared using this solvent, containing cyclohexanol at a concentration of 4.0 mg/mL and chromium (III) acetylacetonate at a concentration of 3.6 mg/mL. The lignin sample was dried under vacuum at 40 °C for at least 24 h. An accurately weighed 25 mg aliquot of the dried lignin sample was placed into a 2 mL glass vial. To this vial, 400 μL of the pyridine/chloroform solvent was added, followed by 150 μL of the internal standard/relaxation agent solution. The mixture was stirred thoroughly to ensure complete dissolution of the lignin. Subsequently, 75 μL of TDMP was added, and the mixture was stirred for approximately 1 min before being rapidly transferred into a 5 mm NMR tube. ^31^P NMR spectrum was acquired with an inverse-gated decoupling pulse sequence using a 25 s pulse delay and 64 scans. Integration windows were set at δ 149–145.6 ppm for aliphatic hydroxyl phosphates, δ 144.4–137.6 ppm for phenolic hydroxyl phosphates, and δ 145.6–144.4 ppm for the internal standard derivative. Chemical shift was calibrated against the signal of the TMDP derivatized cyclohexanol internal standard.

## 3 Results and analysis

### 3.1 Substrates characterization

#### 3.1.1 Chemical composition analysis

The changes in the chemical composition of PL subjected to different pretreatment strategies are presented in Figure 1. Untreated PL comprised 41.2% glucan, 14.5% xylan, and 27.6% lignin. Following DES pretreatment (PL-DES), a glucan recovery rate of 93.2% was achieved, with corresponding xylan and lignin removal rates of 79.2% and 81.1%, respectively. These results indicated that DES pretreatment effectively promoted the fractionation of PL, facilitating substantial removal of hemicellulose and lignin while preserving the majority of cellulose. Nonetheless, the recovery of lignin remained relatively low under this condition. When DES pretreatment was applied to materials that had been subjected to twin-screw extrusion (PL-E-DES), xylan and lignin removal rates increased to 83.1% and 88.1%, respectively, while lignin recovery improved to 71.6%. This enhancement was attributed to mechanical extrusion disrupting the dense fibrous structure of the wood, thereby increasing porosity and improving solvent penetration, which promoted the dissociation of hemicellulose and lignin. Following hydrothermal pretreatment prior to DES (PL-P-DES), lignin recovery further increased to 75.3%, suggesting that hydrothermal treatment enhances lignin recoverability during the subsequent DES process. Notably, the combination of hydrothermal and extrusion pretreatments followed by DES (PL-P-E-DES) achieved the highest lignin removal rate of 89.7% and a lignin recovery of 81.5%. Additionally, analysis of the hydrothermal pretreatment liquor revealed substantial amounts of XOS, corresponding to a total XOS yield of 45.7%. The concentrations of xylobiose (X2), xylotriose (X3), xylotetraose (X4), xylopentaose (X5), and xylohexaose (X6) were 3.84, 4.36, 2.26, 1.55, and 1.27 g/L, respectively. These findings confirmed that mechanical and hydrothermal pretreatments enhanced hemicellulose and lignin dissociation during subsequent DES treatment and significantly improved the recovery of lignin components.

**FIGURE 1:**
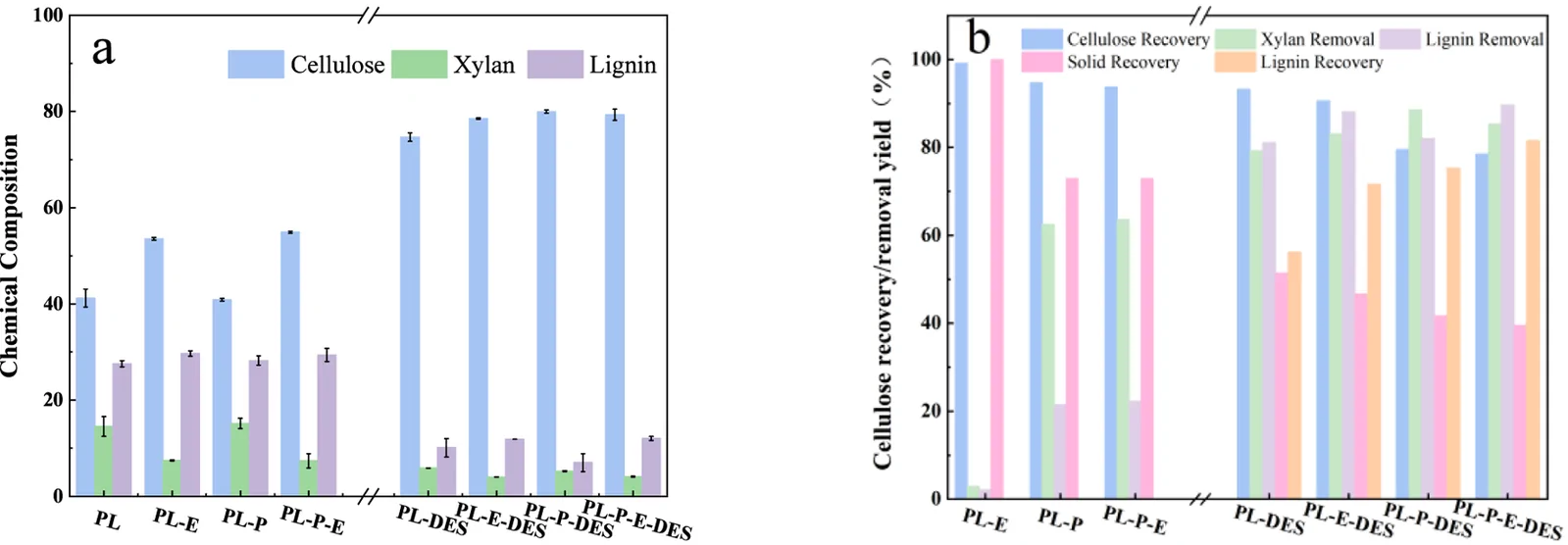
Effect of different pretreatment on the chemical composition **(a)** and recovery/removal yield **(b)**.

#### 3.1.2 Microstructure analysis via SEM

To investigate the effects of various pretreatments on the microstructure of PL, SEM analysis was performed on the resulting residues, as shown in Figure 2. The untreated PL exhibited a smooth and intact surface. In contrast, PL subjected to twin-screw extrusion displayed surface deformation and the formation of large pores within the fibrous structure. Hydrothermal treatment caused the PL to gradually exhibited a broom-like morphology. When twin-screw extrusion was combined with hydrothermal processing, the materials showed pronounced distortion and an increased pore abundance. These observations suggested that extensive hydrolysis of hemicellulose and partial dissociation of lignin led to the depolymerization of the originally dense cell wall structure (Sun et al., 2021). After synergistic DES pretreatment, all samples exhibited varying degrees of structural disruption. With increasing pretreatment intensity, the fiber surfaces transitioned from smooth to highly wrinkled. These results indicated that sequential DES pretreatment further promoted the removal of hemicellulose and lignin. The formation of extensive pore structures is expected to increase the accessible surface area for enzyme-substrate interactions, thereby enhancing the potential for value-added conversion of the pretreated residues.

**FIGURE 2:**
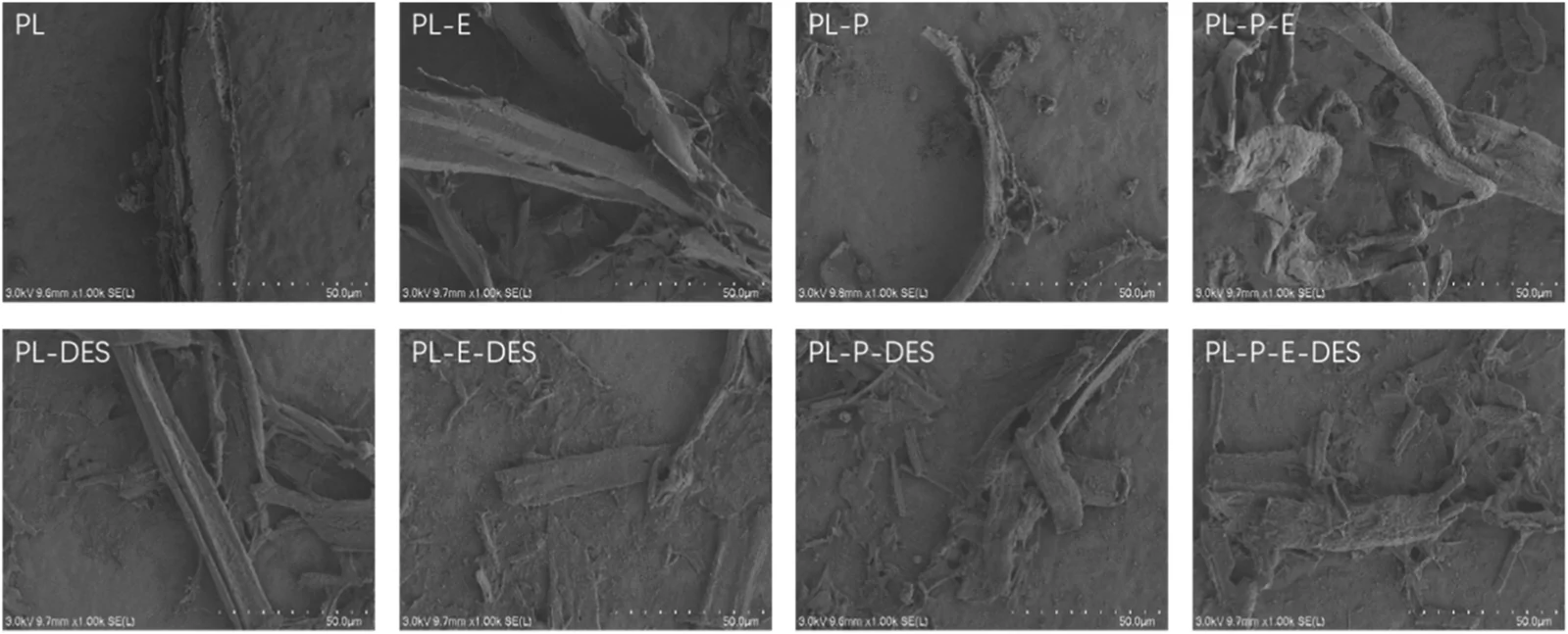
SEM images of different pretreated residues.

#### 3.1.3 XRD analysis of raw and pretreated residues

To investigate the structural changes in fibers resulting from different pretreatments, XRD analysis was performed on the pretreated residues, and the results are presented in Figure 3. Similar to the raw PL, all pretreated samples exhibited distinct diffraction peaks at 2θ = 15.5° and 22.5°, characteristic of the native cellulose I, indicating that none of the pretreatment methods altered the crystalline structure of cellulose. The relative crystallinity of raw PL was calculated to be 44.12%, which increased to 61.80% after hydrothermal treatment (PL-P). This increase was primarily attributed to the hydrolysis of hemicellulose, an amorphous component. Following further delignification in the DES system, the relative crystallinity of PL-DES and PL-P-DES increased to 67.78% and 72.27%, respectively, due to the enhanced removal of amorphous hemicellulose and lignin. Interestingly, the PL-P-E-DES sample exhibited slightly higher xylan and lignin contents compared with the non-extruded PL-P-DES sample, which may be attributable to lignin redeposition on the fiber surface (Huang et al., 2021). This likely accounts for its relatively lower crystallinity of 70.38%.

**FIGURE 3:**
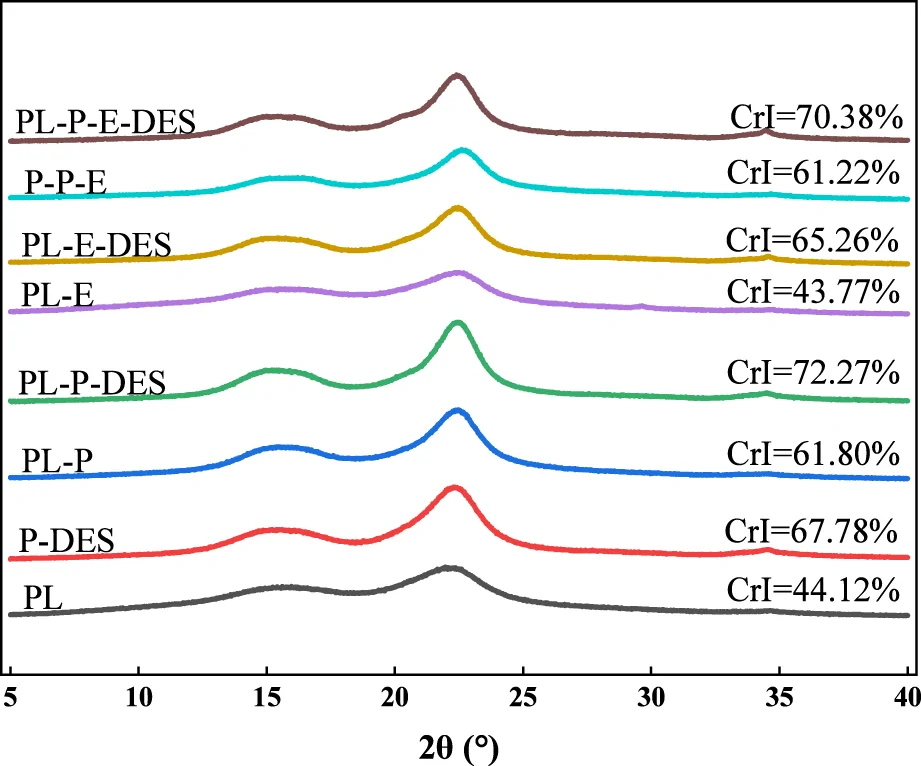
XRD spectrum of different pretreated residues.

#### 3.1.4 FT-IR spectroscopy analysis of raw and pretreated residues

FT-IR spectroscopy was employed to examine the chemical structural changes in biomass following various pretreatments, with the spectra shown in Figure 4. The absorption band areas at 1,427 cm^−1^ and 898 cm^−1^ were analyzed to calculate the cellulose crystallinity index according to the following equation: LOI = A_1427_/A_898._ The LOI value directly reflects the lateral ordering of cellulose chains: A higher LOI corresponds to a greater relative fraction of crystalline domains, more ordered chain packing, and more stable cellulose crystallinity. A lower LOI indicates an increased amorphous fraction, more disordered chain arrangement, and degraded cellulose crystallinity (Jeoh et al., 2024).

**FIGURE 4:**
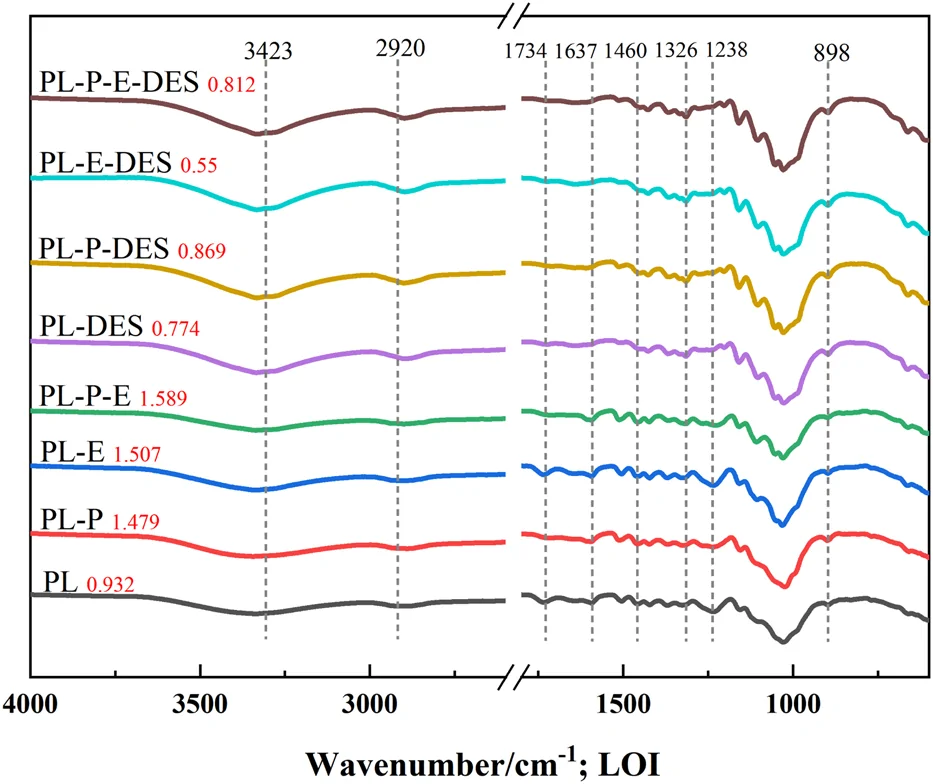
FT-IR image of different pretreated materials, and the red data represent LOI value.

Based on the FT-IR spectra, the calculated LOI values revealed a clear and pronounced trend. The untreated PL exhibited an LOI of 0.93. Following hydrothermal and extrusion pretreatments without DES (PL-P, PL-E, and PL-P-E), the LOI increased to a range of 1.48–1.59. This increase suggested that thermal and mechanical treatments primarily removed amorphous non-cellulosic components, thereby temporarily increasing the relative proportion of ordered cellulose regions. Interestingly, when DES was integrated into the pretreatments (PL-DES, PL-E-DES, and PL-P-E-DES), the LOI values decreased sharply, dropping to a range of 0.55–0.87. This notable reduction clearly indicated that the DES system successfully penetrated the cellulose microfibrils and disrupted intermolecular hydrogen bonds, effectively reducing the lateral ordering and loosening the crystalline structure. This DES-induced structural loosening was highly advantageous, as it exposed more accessible binding sites for cellulase, ultimately boosting enzymatic hydrolysis efficiency.

Compared with the untreated PL, all pretreated samples retained the primary structural absorption peaks. Specifically, absorption bands at 898 cm^−1^, 1,422 cm^−1^, 1,460 cm^−1^, and 1,595 cm^−1^ correspond to typical cellulose structures, while those at 1,238 cm^−1^, 1,326 cm^−1^, 1,506 cm^−1^, and 1,595 cm^−1^ are characteristic of lignin. Peaks observed at 1,637 cm^−1^ and 1734 cm^−1^ are associated with stretching vibrations related to hemicellulose (Yuan et al., 2021). The FT-IR spectra revealed distinct variations in the intensity of these characteristic peaks among the differently pretreated materials. Notably, signals associated with both hemicellulose and lignin diminished following hydrothermal and DES pretreatments, a trend consistent with the compositional analysis results (Shen et al., 2021). Furthermore, the absorption intensities at 3423 cm^-1^ and 2920 cm^-1^ were higher for the PL-DES, PL-P-DES, PL-E-DES, and PL-P-E-DES samples compared with the four residues without DES pretreatment. As reported previously, this enhancement may be attributed to the protective effect of polyols present in the DES system on cellulose, hemicellulose, and lignin. These polyols are suggested to reduce cellulose hydrolysis and react with the Cα position of lignin, thereby protecting its side chains while facilitating lignin dissolution (Li et al., 2024). Consequently, this mechanism leads to the enhanced signal intensities of relevant hydroxyl and cellulose bands in DES-pretreated materials.

### 3.2 Enzymatic hydrolysis

#### 3.2.1 Enzymatic hydrolysis of raw and pretreated residues

The enzymatic hydrolysis yield is a critical metric for assessing the effectiveness of pretreatment strategies. The enzymatic hydrolysis results for various pretreated residues are presented in Figure 5. The glucan hydrolysis yields of PL, PL-E, PL-P, and PL-P-E residues were 18.01%, 16.75%, 24.35%, and 44.25%, respectively. Nonetheless, residual lignin and limited accessibility of binding sites continued to constrain glucan hydrolysis. As demonstrated by Haviland et al. (2024) using single-molecule fluorescence microscopy, residual lignin does not act merely as a nonspecific enzymatic sink; Instead, it forms a physical barrier that explicitly blocks Cel7A access to active binding sites on the cellulose surface and severely impedes the processive movement of the enzymes along the cellulose chains at the nanoscale. Subsequent synergistic pretreatment with a DES markedly improved the enzymatic hydrolysis of glucan across all substrates (Zhang et al., 2025).

**FIGURE 5:**
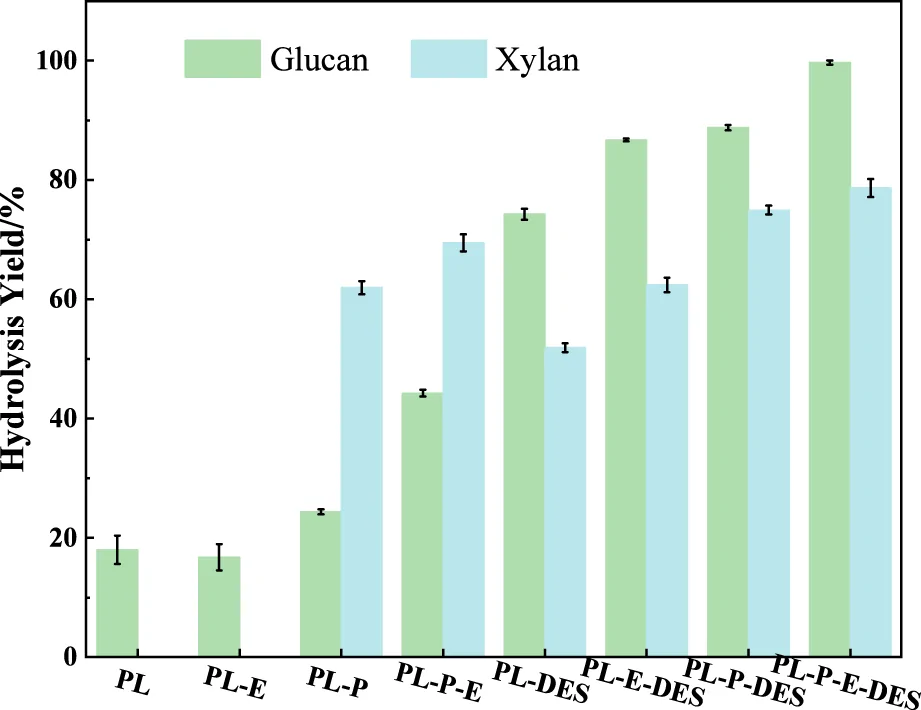
Yield of enzymatic hydrolysis of pretreated materials under different conditions.

The glucan hydrolysis yields for PL-DES, PL-E-DES, PL-P-DES, and PL-P-E-DES reached 74.28%, 86.70%, 88.77%, and 99.69%, respectively. Consistent with the fractionation analysis, DES pretreatment resulted in further extensive removal of hemicellulose and lignin. This enhanced delignification and hemicellulose solubilization increased the specific surface area of the substrate and fully exposed cellulose fibers, thereby significantly improving enzyme-accessible binding sites. As a result, both glucan and xylan hydrolysis yields were substantially elevated. These findings demonstrate that multi-step sequential pretreatment enables efficient fractionation of PL components and facilitated the value-added conversion of cellulose and hemicellulose.

#### 3.2.2 Influence of relevant factors on enzymatic saccharification

To elucidate the mechanisms underlying the enhanced enzymatic hydrolysis (performed with 15 FPU/g cellulase and 300 U/g xylanase) of substrates subjected to hydrothermal (170 °C, 90 min) and DES (100 °C, 30 min) pretreatments, Pearson correlation analysis was conducted between hydrolysis efficiency and key physicochemical properties (Figure 6). As shown in the heat map, enzymatic hydrolysis exhibited a strong positive correlation with both lignin removal (r = 0.80) and xylan removal (r = 0.80). This finding underscored that disruption of the recalcitrant lignin-carbohydrate matrix—via the removal of non-cellulosic barriers—was the predominant driver for improving enzymatic accessibility. Notably, the crystallinity index (CrI) also showed a strong positive correlation with hydrolysis efficiency (r = 0.81) and component removal (r = 0.99). This trend indicated that the observed increases in CrI were primarily attributed to the selective elimination of amorphous hemicellulose and lignin, rather than cellulose recrystallization. Furthermore, the LOI value presented a significant negative correlation (r = −0.77) with hydrolysis yields, which indicated that the DES system successfully penetrated the cellulose microfibrils and disrupted intermolecular hydrogen bonds. These changes led to a loosening of the lateral ordered structure rather than a complete destruction of the crystalline core. Such structural loosening effectively exposed more enzymatic binding sites and, in synergy with the high lignin removal (up to 89.7%), markedly enhanced the glucan hydrolysis yield from 44.25% (PL-P-E) to 99.69% (PL-P-E-DES). In contrast, inadequate lignin removal maintained a higher calculated LOI and physically blocked accessible enzyme-binding sites, thereby exerting a significant inhibitory effect on enzymatic hydrolysis.

**FIGURE 6:**
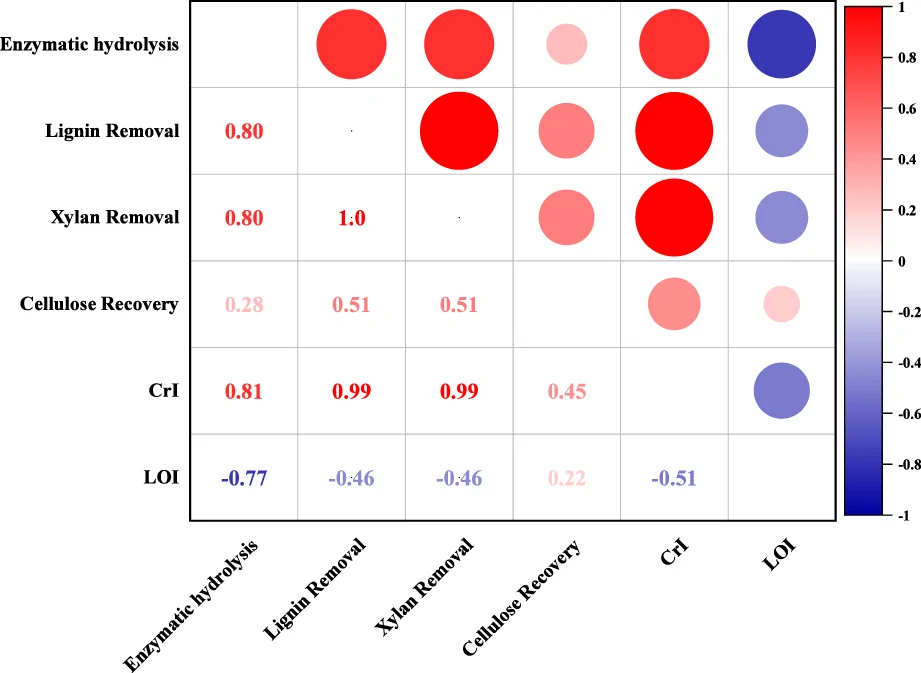
Pearson correlations between substrate properties and enzymatic hydrolysis.

### 3.3 Physicochemical characterization of lignin and its antioxidant properties

#### 3.3.1 FT-IR analysis of lignin

To further elucidate the effect of sequential pretreatment on lignin structure, infrared spectroscopy was employed to characterize the structures of MWL and recovered lignin. As shown in Figure 7, the signal peak at 3423 cm^-1^ originates from the hydroxyl stretching vibration peak in lignin. Compared to the PL-MWL, the hydrothermally treated ground wood lignin (PL-P-E-MWL) sample exhibited a relative increase in the hydroxyl stretching vibration peak. This indicated that after hydrothermal treatment, the removal of hemicellulose led to the breaking of lignin-carbohydrate linkages, exposing more hydroxyl functional groups (Li et al., 2021). After subsequent DES pretreatment, the hydroxyl signals of PL-DES-L and PL-P-E-DES-L further increased, which was potentially associated with extensive lignin depolymerization. The absorption peaks at 2920 cm^−1^ and 2800 cm^−1^ correspond to asymmetric and symmetric C-H stretching vibrations of methyl and methylene groups in side chains.

**FIGURE 7:**
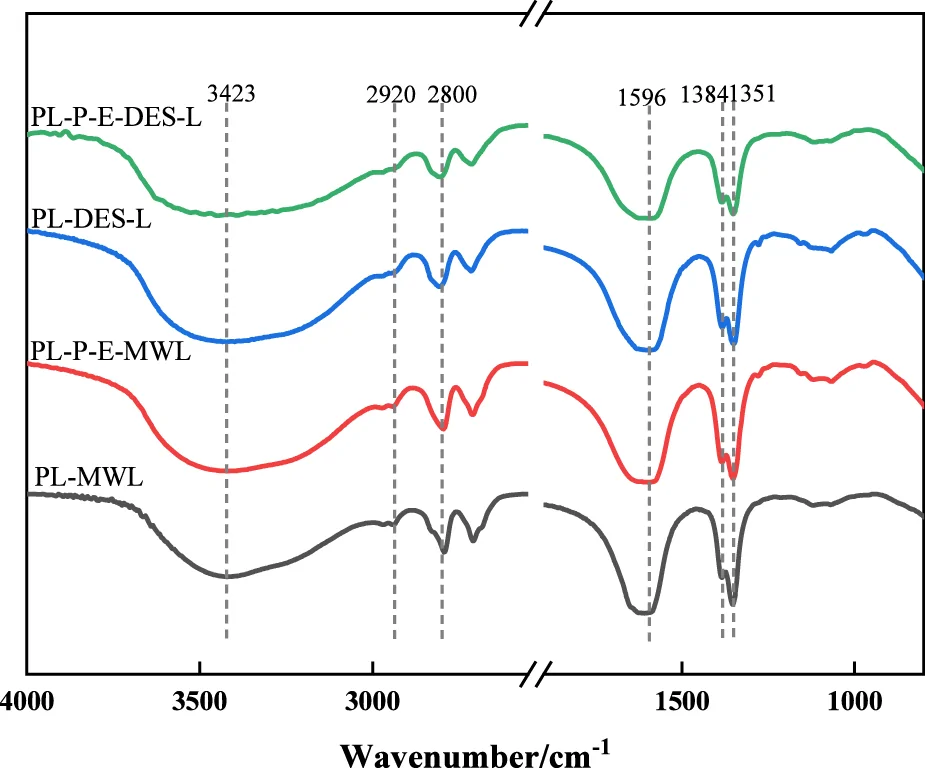
FT-IR image of different lignin samples.

Following DES pretreatment, the intensities of these signals decreased markedly, which could be attributed to the degradation of ferulic acid and *p*-coumaric acid structures in the lignin side chains. In addition, characteristic aromatic ring vibrational absorption of lignin appeared at 1,596 cm^−1^ and 1,384 cm^−1^. The C-O vibration at 1,384 cm^−1^ originated from syringyl (S) unit, whereas the C-O vibration at 1,351 cm^−1^ originated from guaiacyl (G) unit. Notably, in the pretreated lignin, the signal intensity of G units was lower than that of the S units, suggesting that G units are more readily depolymerized than S unit during DES pretreatment. This observation requires further validation by combining lignin molecular weight determination and NMR analysis.

#### 3.3.2 2D HSQC NMR analysis of recovered lignin

In the 2D HSQC NMR spectra, the linkage patterns within the aromatic ring and side-chain regions of lignin can be effectively resolved. Semi-quantitative values for each linkage bond were calculated from the spectra, as shown in Figure 8 and Table 1. In the aromatic region, signals corresponding to syringyl (S), guaiacyl (G), along with minor amounts of *p*-coumaric acid (*p*-CE) and ferulic acid (FA), were clearly observed. The major interunit linkages—β-O-4, β-β, and β-5—accounted for 55.75/100Ar, 10.96/100Ar, and 5.28/100Ar, respectively, yielding an S/G ratio of 1.06. After DES pretreatment, the S unit content increased to 68.79/100Ar, while the G unit decreased to 31.21/100Ar, raising the S/G ratio to 2.20. This indicated that G units were more readily depolymerized than S units during DES pretreatment, implying greater structural stability of S units. Meanwhile, minor polycondensation signals appeared in both S and G units, at 4.04/100Ar and 5.86/100Ar, respectively.

**FIGURE 8:**
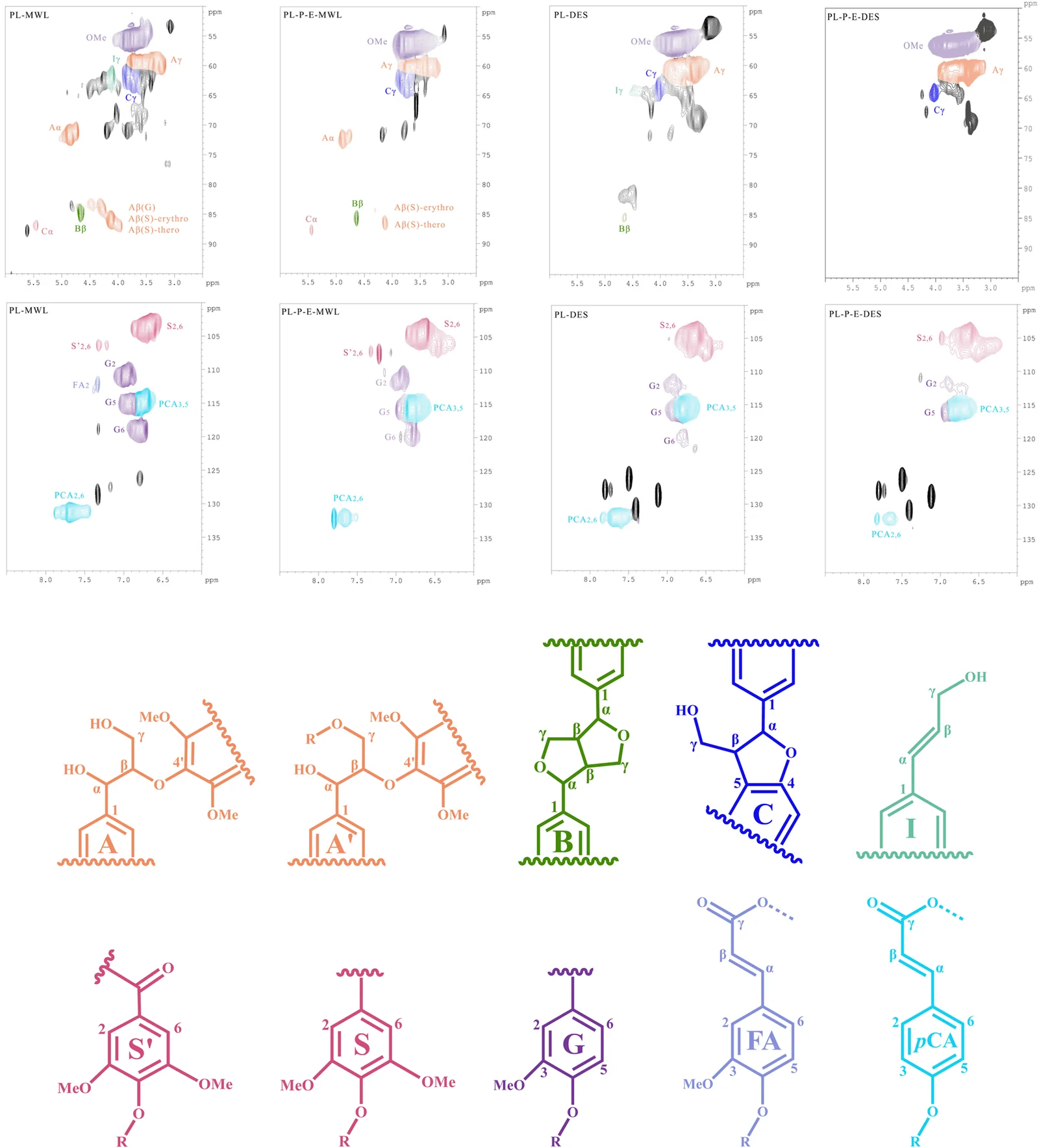
Side-chain and aromatic regions of different pretreated lignin and main chemical structures in the 2D HSQC NMR spectra.

**TABLE 1:** Semi-quantitative content of each junction in lignin (/100Ar).

Sample	PL-MWL	PL-P-E-MWL	PL-DES	PL-P-E-DES
​	Lignin subunits (/100 Ar)			
Syringyl (S)	51.48	65.32 (15.10)	68.79 (4.04)	59.03 (27.27)
Guaiacyl (G)	48.52	34.68 (6.84)	31.21 (5.86)	40.97 (14.27)
S/G	1.06	1.88	2.20	1.44
​	End groups (/100 Ar)			
Ferulic acid (FA)	6.11	2.33	0	0
*p*-Coumaric acid (*p*-CE)	21.56	15.28	21.94	13.11
​	Inter unit linkages (/100 Ar)			
*β- O* -4	55.75	25.87	11.25	0
*β-β*	10.96	4.51	6.01	1.46
*β*-5	5.28	5.06	0	0

In the lignin side-chain region, the ferulic acid signal disappeared, while the coumaric acid content was 21.94/100Ar, suggesting that the side-chain groups of lignin underwent minimal degradation during DES pretreatment. Following hydrothermal treatment of PL, the structure of MWL was partially disrupted. The G unit decreased from 48.52/100Ar to 34.68/100Ar, while the S unit increased to 65.32/100Ar, resulting in an S/G ratio of 1.88. Both S and G units exhibited minor polycondensation signals, with polycondensation ratios of 15.10/100Ar and 6.84/100Ar, respectively. These results indicated that G units were also more prone to depolymerization during hydrothermal treatment, with 61.87% of ferulic acid and 29.13% of *p*-coumaric acid in the lignin side chains undergoing degradation.

Upon subsequent DES pretreatment, the S unit decreased from 65.32/100Ar to 59.03/100Ar, while the G unit increased from 34.68/100Ar to 40.97/100Ar, leading to a decrease in the S/G ratio from 1.88 to 1.44. The signals of the side-chain FA and PCE further decreased, possibly due to a small degree of demethylation of S units, which resulted in an increase in G unit content. Concurrently, the polycondensation signals of S and G units significantly increased to 27.27/100Ar and 14.27/100Ar, respectively. These findings suggested that DES pretreatment of hydrothermally treated materials promoted the formation of lignin polycondensation structures, a phenomenon that warranted further investigation in conjunction with molecular weight measurements.

In the aliphatic region, β-O-4, β-5, and β-β bonds are the primary interunit bonds in lignin. As shown in the table below, direct DES pretreatment led to extensive cleavage of the β-O-4 bond, with its content decreasing from 55.75/100Ar to 11.25/100Ar. The β-β bond content dropped from 10.96/100Ar to 6.01/100Ar, while the β-5 linkage signal disappeared completely. These results indicated substantial depolymerization of lignin during DES pretreatment.

Following hydrothermal treatment of PL, the β-O-4 bond content in MWL decreased from 55.75/100Ar to 25.87/100Ar, and the β-β bond content fell to 4.51/100Ar, indicating that lignin also underwent depolymerization during hydrothermal treatment. Upon subsequent DES pretreatment, the β-O-4 and β-β bond signals vanished, while a β-5 content of 1.46/100Ar remained. This demonstrated that DES pretreatment caused extensive lignin depolymerization, accompanied by pronounced condensation reactions that increased the lignin condensation signal.

It should be emphasized that the 2D-HSQC NMR spectra provide only semi-quantitative relative integral results for lignin interunit linkages. The integral values are suitable for comparative analysis among different samples rather than representing absolute content, which is accompanied by inherent experimental errors.

#### 3.3.3 Molecular weight of lignin

The weight-average (Mw), and number-average (Mn) molecular weight, as well as the polydispersity of lignin are summarized in Table 2. As shown below, the Mw of PL-MWL is 8,126 g·mol^−1^and Mn is 1,536 g·mol^−1^, corresponding to a polydispersity index (PDI) of 5.29. After hydrothermal treatment, the Mw and Mn of PL-P-E-MWL were 7,094 g·mol^−1^ and 2007 g·mol^−1^, respectively, with a PDI of 3.53. Following DES pretreatment, the molecular weight decreased to 1,587 and 878 g·mol^−1^, respectively, yielding a PDI of only 1.81. Combined with the 2D HSQC NMR results of lignin, these data clearly indicated that the acidic DES system significantly promoted lignin depolymerization through the dual effects of hydrogen bond competition and acidic protonation.

**TABLE 2:** The weight-average molecular weight Mw, the number average molecular weight Mn and the dispersity of different lignin.

Sample	M_w_/(g·mol^−1^)	M_n_/(g·mol^−1^)	M_w_/M_n_
PL-MWL	8,126	1,536	5.29
PL-P-E-MWL	7,094	2007	3.53
PL-DES-L	1,587	878	1.81
PL-E-DES-L	1862	927	2.01
PL-P-DES-L	2249	1,022	2.20
PL-P-E-DES-L	2237	1,003	2.23

The recovered lignin exhibited good homogeneity, showing only minor signals from condensed S units (4.04/100Ar) and G units (5.86/100Ar). Further hydrothermal treatment increased the molecular weights of PL-P-DES-L and PL-P-E-DES-L, with their PDI values rising to 2.20 and 2.23, respectively. Semi-quantitative analysis of the linkages in PL-P-E-MWL revealed that after hydrothermal treatment, the contents of β-O-4 and β-β linkages decreased significantly. Concurrently, increased polycondensation signals appeared in the S unit (15.10/100Ar) and G unit (6.84/100Ar).

These results indicated that during hydrothermal treatment, the cleavage of lignin β-O-4 linkages and other bonds generated highly reactive intermediates. These intermediates readily formed C-C bonds with lignin fragments or vacant carbon sites within the lignin matrix, triggering polycondensation reactions. Consequently, the molecular weights of hydrothermally treated lignin samples increased.

#### 3.3.4 Determination of lignin antioxidant properties

The antioxidant properties of lignin samples were evaluated using the DPPH radical scavenging assay. As demonstrated by Zhang et al. in their study on lignin extraction using deep eutectic solvents, the IC50 value—representing the concentration required for a 50% inhibition rate—serves as a critical indicator of radical scavenging capability, where a lower IC50 value inherently signifies a more potent antioxidant activity (Zhang et al., 2024). As shown in Table 3, the positive control Vc exhibited remarkable radical scavenging assay, with an IC50 value of only 35.36 mg/L. The IC50 values of the lignin samples, from lowest to highest, were 56.16 mg/L (PL-E-DES-L), 62.95 mg/L (PL-P-DES-L), 84.41 mg/L (PL-DES-L), and 85.39 mg/L (PL-P-E-DES-L), indicating that all lignin samples possessed good antioxidant properties. Phenolic and free hydroxyl groups are known to promote the antioxidant activity of lignin. Therefore, the hydroxyl content of the lignin samples was characterized via ^31^P NMR.

**TABLE 3:** Hydroxyl content and IC50 value of different lignins.

Sample	aliphatic OH	Syringyl OH	Guaiacyl OH	*p*-coumarate	COOH	Total phenolic OH	IC50 (mg/L)
PL-MWL	4.61	0.05	0.52	0.52	0.06	1.09	-
PL-DES-L	0.80	0.56	0.74	0.45	0.05	1.75	84.41
PL-E-DES-L	1.60	0.87	0.70	0.32	0.08	1.90	56.16
PL-P-E-MWL	2.69	1.13	0.97	0.38	0.33	2.49	-
PL-P-DES-L	1.31	1.09	0.75	0.24	0.07	2.07	62.95
PL-P-E-DES-L	0.91	1.09	0.84	0.40	0.00	2.81	85.39
Vc	-	-	-	-	-	-	35.36

The results showed that PL-MWL and PL-P-E-MWL contained total phenolic hydroxyl groups of 1.09 and 2.49 mmol/g, respectively, while their aliphatic hydroxyl contents decreased from 4.61 to 2.69 mmol/g, consistent with the molecular weight trend described previously. Following DES pretreatment and sequential combination, the aliphatic hydroxyl content in the lignin samples exhibited a decreasing trend, whereas the total phenolic hydroxyl content showed an increasing trend. The total hydroxyl contents, from lowest to highest, were PL-DES-L (2.55 mmol/g), PL-P-DES-L (3.38 mmol/g), PL-E-DES-L (3.49 mmol/g), and PL-P-E-DES-L (3.72 mmol/g). Despite its higher total hydroxyl content, PL-P-DES-L exhibited weaker antioxidant performance than other lignin samples, indicating that multiple factors influence the antioxidant activity of lignin.

Beyond total phenolic hydroxyl content, David and Krishnamurthi reported that the microwave-assisted deconstruction of lignin-carbohydrate complexes (LCCs) significantly accelerates lignin depolymerization, promoting the exposure of reactive functional groups during free radical scavenging, thereby enhancing antioxidant activity (David and Krishnamurthi, 2024). Thus, the relative content of methoxy groups was also a contributing factor. Further analysis of the content of characteristic functional groups is warranted.

## 4 Discussion

In this study, a multi-step sequential pretreatment combining hydrothermal treatment, twin-screw extrusion, and ternary DES treatment was developed for the full-component valorization of poplar biomass. The combined strategy achieved efficient delignification and hemicellulose removal, nearly complete enzymatic saccharification at low enzyme loading, as well as co-production of xylo-oligosaccharides and high-purity antioxidant lignin.

Compared with single-step pretreatment including hydrothermal treatment, twin-screw extrusion, or DES treatment alone, the multi-step process synergistically leveraged mechanical shear, hydrothermal fractionation, and DES delignification. Twin-screw extrusion disrupted the compact cell wall structure, which mitigated lignin redeposition to a certain extent during subsequent hydrothermal processing. Hydrothermal pretreatment removed most hemicellulose in the form of xylo-oligosaccharides. Subsequently, ternary DES further disrupted the intermolecular hydrogen bonds of cellulose and removed residual lignin, providing sufficient accessible sites for cellulase. Pearson correlation analysis confirmed that delignification and xylan removal were the dominant factors determining enzymatic hydrolysis efficiency, whereas increased crystallinity mainly originated from the elimination of amorphous fractions. Meanwhile, DES cleaved the β-O-4 linkages of lignin, while hydrothermal treatment induced partial lignin condensation. The obtained lignin possessed a high phenolic hydroxyl content and exhibited favorable DPPH radical scavenging activity.

Nevertheless, this work still has several limitations with respect to practical application. First, the recyclability of the ternary DES system was not investigated in the present study. Although related reports have demonstrated that choline chloride-based DES can be recovered via simple evaporation, repeated recycling may cause component loss, degradation of acid groups, and performance attenuation, which would increase the overall process cost. Therefore, solvent recovery, stability, and economic evaluation should be prioritized in our follow-up investigation. Second, the antioxidant capacity of recovered lignin was only evaluated by the DPPH radical scavenging assay. It should be noted that a single antioxidant evaluation model cannot fully reflect the comprehensive antioxidant performance of lignin. Additional assays such as ABTS or FRAP are required for cross-validation in future work. In addition, all experiments were performed on poplar wood, and the applicability of this integrated pretreatment to other types of lignocellulosic feedstocks remains to be verified.

Future work can be carried out from the following aspects: (1) optimize twin-screw extrusion parameters including rotation speed and feed moisture to further reduce energy consumption; (2) explore the recovery and recycling performance of the ternary DES solvent and carry out a preliminary techno-economic assessment; (3) expand feedstocks to other hardwoods, softwoods, or agricultural residues to verify the universality of this multi-step sequential pretreatment strategy; and (4) adopt multiple antioxidant evaluation systems to comprehensively characterize the antioxidant properties of the isolated lignin.

## 5 Conclusion

This study developed single-step and multi-step synergistic pretreatment strategies for the integrated valorization of poplar biomass into fermentable monosaccharides, functional oligosaccharides, and antioxidant lignin derivatives, thereby advancing the sustainability of lignocellulosic biorefineries. Chemical composition analysis demonstrated that the multi-step sequential pretreatment achieved efficient fractionation of poplar, yielding a lignin removal of 89.7%, a lignin recovery of 81.5%, and an XOS yield of 45.7%, indicating superior biomass fractionation compared with single-step pretreatment. Consequently, the removal of structural barriers remarkably enhanced enzymatic hydrolysis efficiency, with the enzymatic hydrolysis yields of glucan and xylan reaching 99.7% and 78.7%, respectively. Furthermore, the recovered lignin exhibited a high phenolic hydroxyl content of 2.81 mmol/g and a low IC50 value of 56.16 mg/L, suggesting excellent antioxidant activity. These findings provide a solid foundation for the high-value utilization of lignin in biorefinery applications.

## Data Availability

The original contributions presented in the study are included in the article/supplementary material, further inquiries can be directed to the corresponding authors.
